# Climate warming and drought modify galling effects on tall goldenrod

**DOI:** 10.1007/s00442-026-05889-3

**Published:** 2026-04-04

**Authors:** Emily G. Parker, Kara C. Dobson, Moriah L. Young, Mark D. Hammond, Phoebe L. Zarnetske

**Affiliations:** 1 W. K. Kellogg Biological Station, Michigan State University, Hickory Corners, MI USA; 2 Department of Integrative Biology, Michigan State University, East Lansing, MI USA; 3 Ecology, Evolution, and Behavior Program, Michigan State University, East Lansing, MI USA

**Keywords:** Climate change, Multiple stressors, Plant–insect interactions, Herbivory, *Solidago altissima*

## Abstract

Plants must respond to changing climatic conditions while continuing to defend against herbivores. While numerous studies have investigated how one type of stress affects plants, the effects of multiple abiotic and biotic stressors and their interactions are less understood. We used the Rainfall Exclusion eXperiment (REX) at the Kellogg Biological Station Long-Term Ecological Research site (KBS LTER) to quantify the individual and interactive effects of drought and warming treatments (abiotic stressors), and galling by *Rhopalomyia solidaginis* (biotic stress) on tall goldenrod (*Solidago altissima*), a common native plant species in Michigan, USA. At the end of the 2021 and 2022 growing seasons, we measured stem height and biomass as a proxy for plant productivity, and seed mass per stem as a proxy for reproductive fitness. We also measured gall biomass, larval chamber number, and larval chamber volume to reflect the effects of drought and warming on the gallmaker. We found that warming mitigated some negative galling effects; galled plants were 7.1 cm shorter than non-galled plants in ambient conditions, but under warming, there was no reduction in height for galled plants. Furthermore, drought exacerbated some galling effects: galled plants experiencing drought conditions had the lowest probability of producing seeds (0.47) compared to plants from all other treatments. Understanding how plants respond to individual abiotic and biotic stressors as well as their interactions will enhance our ability to predict plant fitness and community dynamics under new climate regimes.

**Supplementary Information:**

The online version contains supplementary material available at 10.1007/s00442-026-05889-3.

## Introduction

Plants can respond in a variety of ways to climate change (Parmesan and Hanley 2015). Climate warming typically advances plant phenological events and increases plant size, both of which have varying implications for plant fitness (Willis et al. 2008; Springate and Kover 2014; Anderson 2016; Wangchuk et al. 2021). For example, earlier flowering has been linked to larger plant size, resulting in higher individual fitness (Springate and Kover 2014). However, other traits more directly linked with plant fitness, such as flower and seed production, show variable responses to warming; reproductive output can increase or decrease depending on factors such as plant species type (e.g., trees vs. shrubs vs. forbs vs. graminoids) and the magnitude of warming (Dobson and Zarnetske 2025). In contrast, drought generally reduces plant fitness by decreasing growth and productivity, leaf surface area, and reproductive output (Farooq et al. 2012). In addition to these shifting climate conditions, plants must also fend off biological stressors such as herbivores, diseases, and parasites (Garrett et al. 2006; Hamann et al. 2021; Liu et al. 2024). These dual pressures often demand conflicting survival strategies, in which a plant’s defense mechanisms may come at the cost of growth or reproduction, especially under various climate stressors (Coley et al. 1985; Agrawal 2007; Pellissier et al. 2018).

One common biotic pressure plants face is herbivory, which affects plant productivity, phenology, and chemical composition (Ritchie et al. 1998; Post and Pedersen 2008; Lemoine et al. 2017). In future climate scenarios, herbivory pressure is predicted to increase for herbaceous plants worldwide (Liu et al. 2024). Climate stress can also influence plant responses to herbivory (Lemoine et al. 2017; Kaarlejärvi et al. 2017; Young and Dobson et al. 2024). For example, Lemoine et al. (2017) found that under warmed conditions, the negative effects of herbivory on plant height, as well as the positive effects of herbivory on fruit mass, were greatly reduced. Galling is a specialized form of parasitic herbivory that involves insects ovipositing eggs into plant tissue, spurring the growth and development of a gall (Raman 2011). Within the gall, the larval insect will develop, receiving nutrients and protection from unfavorable environmental conditions from its plant host (Price et al. 1987). Galling by insects has been found to reduce the host plant’s reproductive fitness, shift nutrients away from other plant structures, and alter plant architecture (Hartnett and Abrahamson 1979; Florentine et al. 2005; Crutsinger et al. 2008). While the gall shields developing larvae from environmental stress, gallmakers are not immune to climate stressors. Drought can lead to extirpation of gallmakers and reduced gall size (Sumerford et al. 2000; Björkman 2000). In a study investigating warming and drought effects on gallmakers, warming increased insect mortality under decreased precipitation, but did not affect survival under normal or increased precipitation regimes (Xi et al. 2016). In sum, although previous studies have investigated the effects of climate and insect herbivory on plants, our understanding of the interactions among climate stressors and gall-specific herbivory stress on plant fitness remains limited.

Goldenrods (genus *Solidago*) are common forb species in North American old-field communities (Abrahamson et al. 2005; Perez et al. 2024) and play important roles in early successional communities across their range, supporting higher trophic levels by providing resources for pollinators, herbivores, and gallmakers (Hartnett and Abrahamson 1979; Werner et al. 1980). Goldenrods have broad climatic tolerances, multiple reproductive strategies, and allelopathic effects on other flora, and these traits allow goldenrods to dominate plant communities across Canada and the United States (Werner et al. 1980; Perez et al. 2024), as well as in their introduced range of Europe and Asia (Szymura and Szymura 2016; Szymura et al. 2018). *Solidago spp.* can reduce total plant species diversity and evenness through competitive exclusion and shading of lower stature plants, as well as through allelopathic chemical compounds (Fisher et al. 1978; Schmitz 2003; Souza et al. 2011). In their native range, *Solidago spp.* are susceptible to attack by several species of gallmakers; some gallmakers specialize on a single species of goldenrod, while others may attack many species of goldenrod (Dorchin et al. 2009). In terms of climate effects on goldenrod, Rosenblatt (2018, 2021) found that within the same system and same experimental setup, warming may have negative impacts on *S. rugosa* survival one year, but not the next year. Multiple studies have shown that water stress (drought) decreases vegetative and reproductive biomass in multiple goldenrod species (*S. gigantea* and *S. altissima*: Shibel and Heard 2016; *S. rugosa*: Rosenblatt 2018). The combination of warming and drought has been shown to interact additively, decreasing inflorescence presence and individual survival in *S. rugosa* (Rosenblatt 2018, 2021). There are few studies on how goldenrods respond to co-occurring abiotic and biotic stress. Clipping (simulated herbivory) and water stress have been shown to synergistically reduce *S. altissima* and *S. gigantea* aboveground biomass and inflorescence production (Shibel and Heard 2016). In our old-field system, goldenrod biomass increases with warming (Young and Dobson et al. 2024) and is resilient to drought events (Perez et al. 2024). However, to our knowledge, no study has investigated goldenrod’s responses to warming, drought, and galling together.

In this study, we investigated how tall goldenrod (*S. altissima*) responded to multiple abiotic conditions (warming and drought) and an important biotic interaction (galling). In an old-field plant community at the Kellogg Biological Station Long-Term Ecological Research Site (KBS LTER) in Michigan, USA, we manipulated the abiotic environment by deploying open-top chambers (OTCs) to induce warmer temperatures and rainout shelters to induce drought in a fully factorial design over two years. We measured plant productivity traits (stem height, stem biomass), and reproductive traits (seed mass) for stems with and without *Rhopalomyia solidaginis* galls. We also measured gall biomass and larval chamber count and volume to quantify potential climate effects on the insects’ fitness. Our hypotheses are as follows:

### Hypothesis 1

*The positive effects of warming will mitigate the negative effects of galling*.

Warming alone will positively impact individual stem fitness and productivity via increased stem height, biomass, and seed production (Molau 1997; De Frenne et al. 2011; Young and Dobson et al. 2024; Dobson and Zarnetske 2025). This will mitigate the negative effects of galling (Hartnett and Abrahamson 1979).

### Hypothesis 2

*Drought and galling will synergistically reduce plant fitness.*

Drought alone will reduce individual stem productivity and reproductive fitness (Rosenblatt 2018, 2021). Galling presence on stems will enhance abiotic stress and further reduce productivity and reproductive fitness (Shibel and Heard 2016).

### Hypothesis 3

*Warming will reduce the negative effects of drought and galling.*

Stems with galls experiencing the combination of warming and drought will have lower fitness compared to stems with galls experiencing only warming, but higher fitness compared to stems with galls experiencing only drought. Similarly, stems without galls experiencing warming and drought will have lower fitness than stems without galls experiencing warming alone, but higher fitness than stems without galls experiencing drought alone.

### Hypothesis 4

*Drought and warming will reduce gallmaker fitness* via *gall size.*

Galls experiencing warming and drought will be smaller than galls in ambient conditions, with smaller larval chambers due to lower insect fitness (Sumerford et al. 2000; Björkman 2000; Xi et al. 2016).

## Methods

### Site description

This study was conducted at the Kellogg Biological Station Long Term Ecological Site Main Cropping System Experiment (KBS LTER MCSE) located in Hickory Corners, MI, USA (42.41°N, 85.37°W). The KBS LTER is located in a temperate climate with a mean annual temperature of 10.1 °C (30 year average, 1981–2011) and mean precipitation of 100.5 cm (Robertson and Hamilton 2015). The soils of the KBS LTER are well-drained Alfisol loams, with surface soils containing an average of 17% clay and 43% sand content (Crum and Collins 1995; Robertson and Hamilton 2015). Our experimental plots were located in six early-successional community field replicates (1 ha each). When agricultural management of these fields ended in 1988, an early-successional plant community was reestablished and has been minimally managed, with an annual spring burn and sporadic herbicide applications to shrub and tree species (ex. black locust, *Robinia pseudoacacia*) to maintain a diverse grassland ecosystem. This plant community is composed of non-agronomic forb, grass, and legume species, and is commonly referred to as an “old field.” *Solidago altissima* (historically called *Solidago canadensis* in this system; see supplement for identification notes) is the dominant species in this community (Perez et al. 2024). *S. altissima* is susceptible to attacks by several species of gallmakers (Hartnett and Abrahamson 1979), and galling by *Rhopalomyia solidaginis* on *S. altissima* stems is a frequent occurrence at the KBS LTER (Howard et al. 2025). Due to goldenrod’s ability to reproduce clonally and our inability to tell if two stems are genetically identical ramets, we refer to individuals as “stems” for this study.

### Insect identification

*Rhopalomyia solidaginis* Loew 1862 (family: Cecidomyiidae, Diptera) is a gallmaker of multiple goldenrod species in eastern North America, mainly *Solidago altissima* (Dorchin et al. 2009). This species is bivoltine, with one generation occurring in early spring (April–May) and another later in the summer (June–September). The second generation produces characteristic “rosette” galls on the apical leaf bud of goldenrod stems, and contains 1–12 larval chambers which each house and protect a single developing larva (Raman and Abrahamson 1995; Dorchin et al. 2009). Galls were identified visually based on descriptions and images from Dorchin et al. (2009) and host plant identity.

### Experimental design

**Fig. 1:**
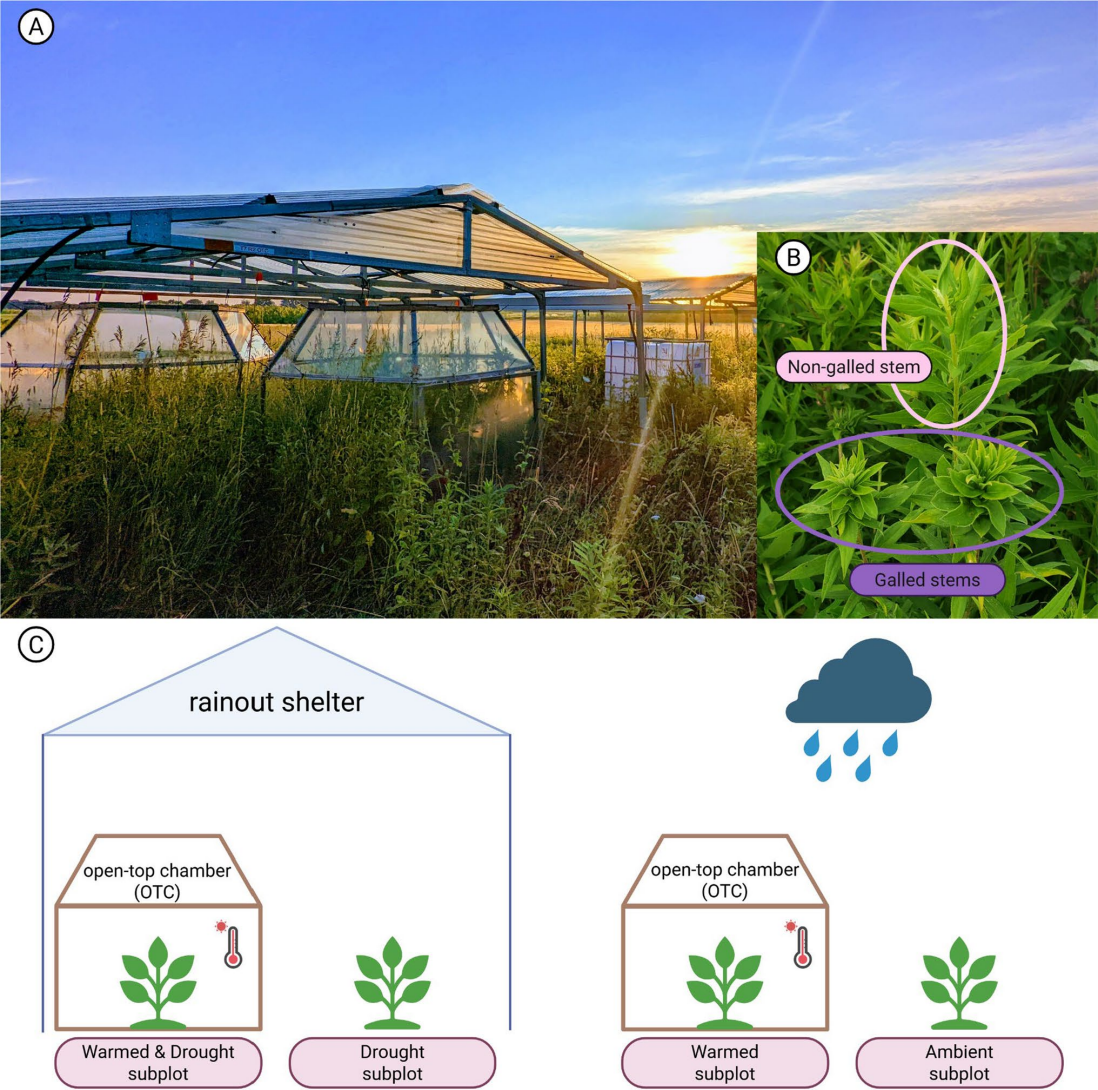
**A** Photograph of the experimental setup of drought and warming subplots (photo credit: Kara Dobson), **B** a close-up photograph of non-galled (top pink circle) and galled (bottom purple circle) tall goldenrod (*Solidago altissima*) stems, and **C** a simplified diagram representing the experimental design. Each field replicate (*n* = 6; one example field replicate is shown in the figure) contains a drought (rainout shelter) and a non-drought (raincloud) plot. Within each plot, subplots receive either an OTC for warming or no OTC. Each field replicate therefore contains four climate treatments: warming and drought combined, drought, warming, and an ambient control subplot. Created in https://BioRender.com

The four climate treatments used in this study were warmed, drought, warmed & drought, and ambient (control). To investigate how early-successional plant communities respond to drought and warming stress, the Rainfall Exclusion eXperiment (REX) was deployed within each early-successional field replicate (*n* = 6) in 4.25 × 5.5 m plots (*n* = 2 per field replicate), each containing 1 m^2^ subplots (*n* = 2 per plot) in the 2021 and 2022 growing seasons at the KBS LTER MCSE (Fig. 1c). Each of the six field replicates contained all four climate treatments for a total of 24 subplots (6 field replicates × 4 treatments per field replicate, Fig. 1).

Within each field replicate, one plot received a rainout shelter, which allows for the manipulation of drought conditions. Shelters consist of a galvanized steel frame with a plexiglass (Lexan) roof to allow sunlight to pass through and gutters to redirect rainfall away from the sheltered area (Fig. 1). Specifics of shelter design are described by Kahmark et al. (2024). Rainfall was excluded from the drought plots (drought, warmed & drought subplots) for 6 weeks from mid-June to August, when soils in the region are the most prone to drought (S. Hamilton and N. Baker, pers. comm.). Two weeks prior to the initiation of drought, shelters were placed over the drought plots for two weeks and watered weekly to the 30 year average rainfall amount (2 cm) to equilibrate initial soil moisture conditions among sheltered plots. A 2-cm ‘re-wetting’ event ended the drought before shelters were removed for the rest of the year. In 2021, the drought started on 19-Jul and ended 27-Aug. For 2022, the drought started on 25-Jun and ended 05-Aug. Non-drought plots (ambient and warmed subplots) did not receive a rainout shelter overhead and received natural levels of precipitation during this time.

Temperature was manipulated using tall-stature open-top chambers (OTCs) installed over one 1 m^2^ subplot within each plot as a warming treatment (see Welshofer et al. 2018b for OTC design; Fig. 1c). The OTCs passively increase air temperatures while allowing for natural levels of light, precipitation, and gas exchange to occur (Marion et al. 1997; Welshofer et al. 2018). The rainout shelters were tall enough to nest OTCs underneath, allowing for both warming and drought to be applied to subplots simultaneously (Fig. 1). OTCs were left in the field year-round except for 1–2 weeks in early spring (usually March/early April) when they were removed from the subplots for prescribed burns of the entire early-successional field sites (Robertson and Hamilton 2015).

Goldenrod stems found inside the subplots were tagged in early July as galls were beginning to form. One to five galled and non-galled stems (depending on the number of stems present in the subplot) were haphazardly selected in each treatment subplot in each replicate, for a total of approximately 240 stems per year of the experiment (see supplementary table S1 for the number of stems per treatment per year). The initial experiment took place in 2021, and was repeated in 2022 for a total of two years of data collection.

### Monitoring of abiotic conditions

Hourly air temperature was recorded at the subplot-level, one meter aboveground, using HOBO pendants (MX2202, Onset Computer Corporation, Bourne, MA). Plastic dish solar shields were installed above each air temperature sensor to mitigate the impact of solar radiation on air temperature readings. A single pendant was placed in spring 2021 in each of the 24 subplots, except in the drought and warmed & drought subplots, where they were not placed until September of that year. Due to their late deployment, the air temperature data from the drought and warmed & drought treatments for 2021 were removed from analyses. Soil moisture and temperature were monitored using Campbell Scientific probes (CS650 SDI-12) and data loggers (CR1000). Probes were installed in July 2021, and were 30 cm in length and installed at 54 degrees from the soil surface to yield soil-surface temperature (Kahmark et al. 2024). A single probe was placed in most of the 24 subplots, though some probes were lost due to animal and accidental human damage. One ambient and one warmed subplot were too far from the monitoring station to place a probe, so they did not have soil monitoring data for either year. All abiotic measurements were collected during the growing season (July–October) of both years, with the exceptions listed above.

### Data collection

The plant traits collected were: stem height and biomass (measures of individual stem productivity) and seed mass (reproductive productivity/fecundity). We also measured gall biomass, larval chamber count, and larval chamber volume to assess the effects of climate on insect fitness (Raman and Abrahamson 1995). Individual stems were clipped at ground level at the end of the growing season, when all seeds on the inflorescence were mature (~ early to mid-October). Height was measured to the tallest point of the plant, including galls and inflorescences if present. For example, if a stem had an inflorescence, the height would be measured to the top of the inflorescence. Stems without an inflorescence were measured to the top of the stem or gall (if present). Galls and inflorescences were removed from the stem if present to obtain individual stem biomass measurements. In 2021, galls were dissected to expose larval chambers to assess severity of galling. Larval chambers were counted, and their diameter and height were measured to calculate chamber volume (see Raman and Abrahamson 1995 methods and Fig. 1). Following dissection, galls, remaining stems, and associated leaves were placed in a drying oven at 60 °C for 3–4 days. Once dry, stems and galls were weighed for biomass measurements. The inflorescences were air dried and processed by hand in the lab to yield only seeds (achene & pappus). Specifically, seed removal was accomplished by manually rubbing the heads (capitulum) of the inflorescence to release the seeds from the involucral bracts and then using dissecting forceps to separate the seed material from other material such as bracts (Gross and Werner 1983). These air dried seeds were then weighed as a proxy of reproductive fitness.

### Statistical analyses

All analyses were conducted using R version 4.4.1 (R Core Team 2024). To test for the effects of climate treatment and galling, we ran separate linear mixed models for each response variable using the R package lmerTest (Kuznetsova et al. 2017; R Core Team 2024). The model included the fixed effects of climate treatment and galling status, and the random effects of year and subplot nested within plot nested within field replicate to account for the hierarchical structure of the experimental design: Response variable_i_ = *β*_0_ + *β*_1_Climate_Treatment_*i*_ + *β*_2_Galling_Status_*i*_ + *β*_3_(Climate_Treatment × Galling_Status)_*i*_ + *α*_Rep[*i*]_ + *α*_Plot[*i*]_ + *α*_Subplot[*i*]_ + *α*_Year[*i*]_ + *ϵ*_*i*_*; α​∼N(0,σ*_*α*_^*​2*^*​)*. Gall-specific variables (i.e., gall mass, chamber count, and chamber volume) used the same model structure, but without the galling status effect. For each response variable, we tested for an interaction between climate treatment and galling status. A significant interaction term would demonstrate that the effect of galling depends on the climate treatment of the stem. We then checked all pairwise treatment comparisons using the emmeans package (Lenth 2022).

We confirmed the data fit the assumption of normality prior to running our models and that there were no outliers with Bonferroni-adjusted outlier tests*.* Stem height was transformed using a log transformation, and stem biomass and gall mass were transformed using a square-root transformation. Because the seed-mass data contained an excess of zeros, we performed a zero-inflated gamma model using the glmmTMB package (Brooks et al. 2017). Using this model, we evaluated the probability of a stem producing at least one seed (a binomial response), and if seeds were produced, the total seed mass produced (a truncated negative-binomial response). In this model, random effects included the nested effects of plot, replicate, and year, as well as a separate random effect for stem biomass; this controlled for the total biomass of the stem when analyzing total seed mass and the probability of producing a seed.

## Results

### Abiotic conditions

**Fig. 2:**
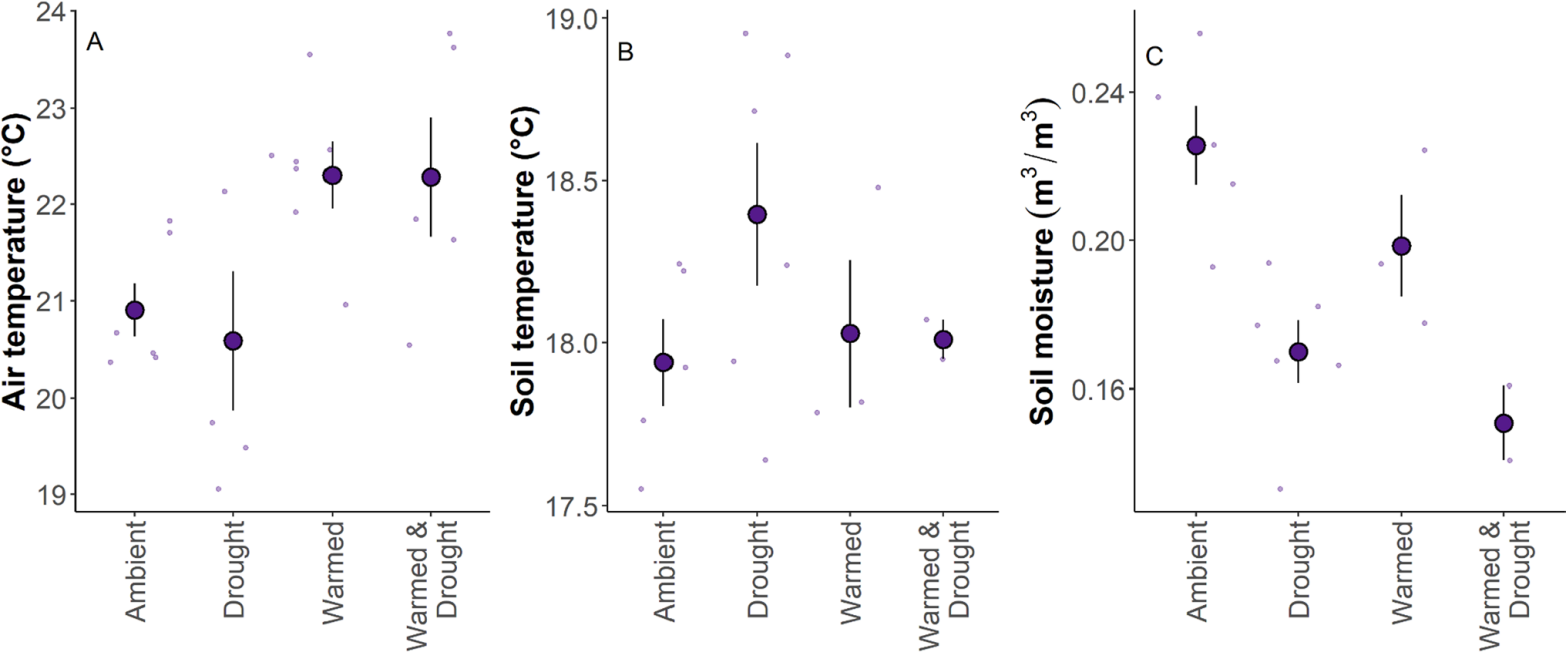
**A** Air temperature (°C) at 1 m aboveground, **B** soil temperature (°C) at an integrated depth of 25 cm belowground, and **C** soil moisture (m3/m3) at an integrated depth of 25 cm belowground across all climate treatments (ambient, warmed, drought, and warmed & drought). Jittered points in the background represent average measurements per plot. Large points represent mean ± standard error. Air temperature sample sizes: Ambient and Warmed *n* = 6, Drought and Warmed & Drought *n* = 5. Soil temperature and moisture sample sizes: Drought *n* = 6, Ambient *n* = 5, Warmed *n* = 3, Warmed & Drought *n* = 2. Differences in the number of sensors are due to failed sensors, lack of sensors due to excessive distance from logger, or soil sensors being eaten by small mammals or accidentally clipped/cut while soil coring or plant harvesting

From July to October, air temperatures in the warmed treatment were 1.2–1.4 °C hotter than the ambient (*z* = −6.26, *P* < 0.0001) and drought (*z* = −4.22, *P* = 0.0001) treatments (Fig. 2A). The warmed & drought treatment’s air temperatures were 1.7 °C hotter than air temperatures in the drought treatment (*z *= −4.96, *P* < 0.0001). The drought treatment appears to have the hottest soil temperatures (Fig. 2B); however, there were no significant differences in surface soil temperatures between climate treatments. The drought and warmed & drought treatments had 0.04–0.07 m^3^/m^3^ less soil moisture than the ambient (drought: *z* = 18.2, *P* < 0.0001; warmed & drought*:* *z* = 14.9, *P* < 0.0001) and warmed (drought: *z* = −9.37, *P* < 0.0001; warmed & drought: *z* = 9.90, *P* < 0.0001) treatments (Fig. 2C).

### Stem biomass and height

**Fig. 3:**
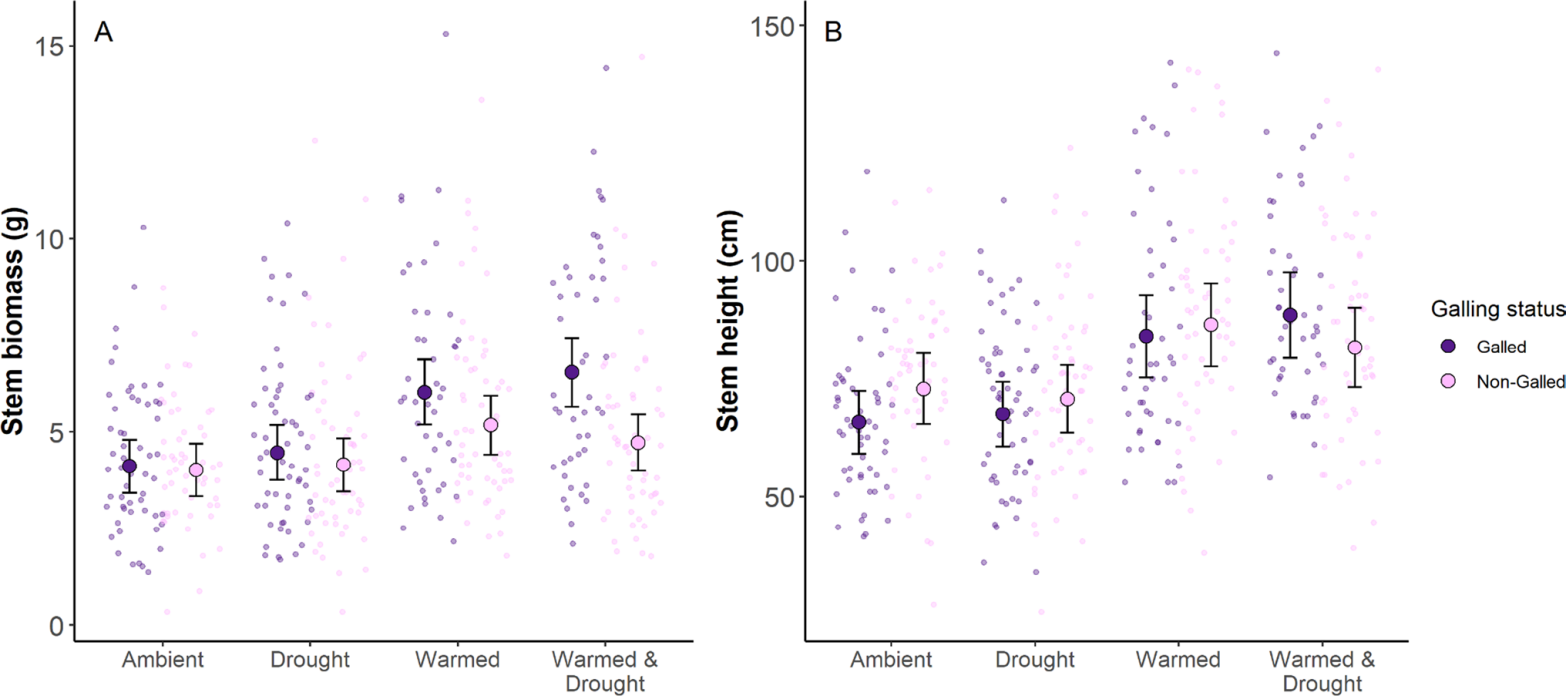
**A** Dried stem biomass (*g*) and **B** height for stems in each climate treatment (ambient, drought, warmed, and warmed & drought). Data are split within each climate treatment for stems with galls (purple) and stems without galls (pink). Jittered points in the background represent stem biomass for individual stems from each treatment. Large points and error bars represent the model-estimated mean ± standard error. Sample sizes are in Table S1

The effect of galling on stem biomass differed with climate treatment (climate x galling; *F*_34141_ = 3.43, *P* = 0.02; Fig. 3A). In the ambient and drought treatments, galling did not significantly affect biomass (ambient: *t*_414_ = 0.29, *P* = 0.77; drought: *t*_410_ = 0.92, *P* = 0.36). However, in the warmed treatment, galled stems had 16% greater biomass compared to non-galled stems (*t*_414_ = 2.07, *P* = 0.04). This effect was strongest in the warmed-and-drought treatment, where galled stems had 39% greater biomass than non-galled stems (*t*_424_ = 4.30, *P* < 0.0001). Among galled stems, biomass was 47% higher in the warmed treatment (*t*_32_ = −3.72, *P* = 0.004) and 59% higher in the warmed-and-drought treatment (*t*_26_ = −3.87, *P* = 0.003) compared to the ambient treatment. Similarly, biomass of galled stems was 46% greater in the warmed treatment (*t*_25_ = −2.54, *P* = 0.08) and 55% greater in the warmed-and-drought treatment (*t*_33_ = −3.82, *P* = 0.003) compared to the drought treatment. Among non-galled stems, only in the warmed treatment were there marginal differences in biomass compared to the ambient control (*t*_29_ = −2.43, *P* = 0.09). These results were consistent across both years of the study (Fig. S2).

The effect of galling on stem height differed with climate treatment (climate x galling: *F*_3410_ = 3.42, *P* = 0.02; Fig. 3B). Galled stems were 7.1 cm shorter than non-galled stems in the ambient treatment (*t*_412_ = −2.54, *P* = 0.01) but 6.9 cm taller in the warmed-and-drought treatment (*t*_418_ = 1.89, *P* = 0.06). Furthermore, galled stems in the warmed and warmed-and-drought treatments were taller than galled stems in the ambient (warmed: 18.2 cm taller, t_23_ = −3.39, *P* = 0.01; warmed-and-drought: 22.7 cm taller, *t*_22_ = −3.54, *P* = 0.009) and drought (warmed: 16.5 cm taller, *t*_21_ = −2.63, *P* = 0.07; warmed-and-drought: 21.0 cm taller, *t*_23_ = −3.74, *P* = 0.005) treatments. However, there were no differences in stem height between climate treatments for non-galled stems (Table S3). These results were consistent across both years of the study (Fig. S3).

### Seed mass and probability

There was an interactive effect of climate treatment and galling on the probability of producing a seed and on seed mass (climate x galling: *X*^2^ = 11.0, df = 3, *P* = 0.01; Fig. 5; Table S4).

#### Probability of producing a seed

**Fig. 4:**
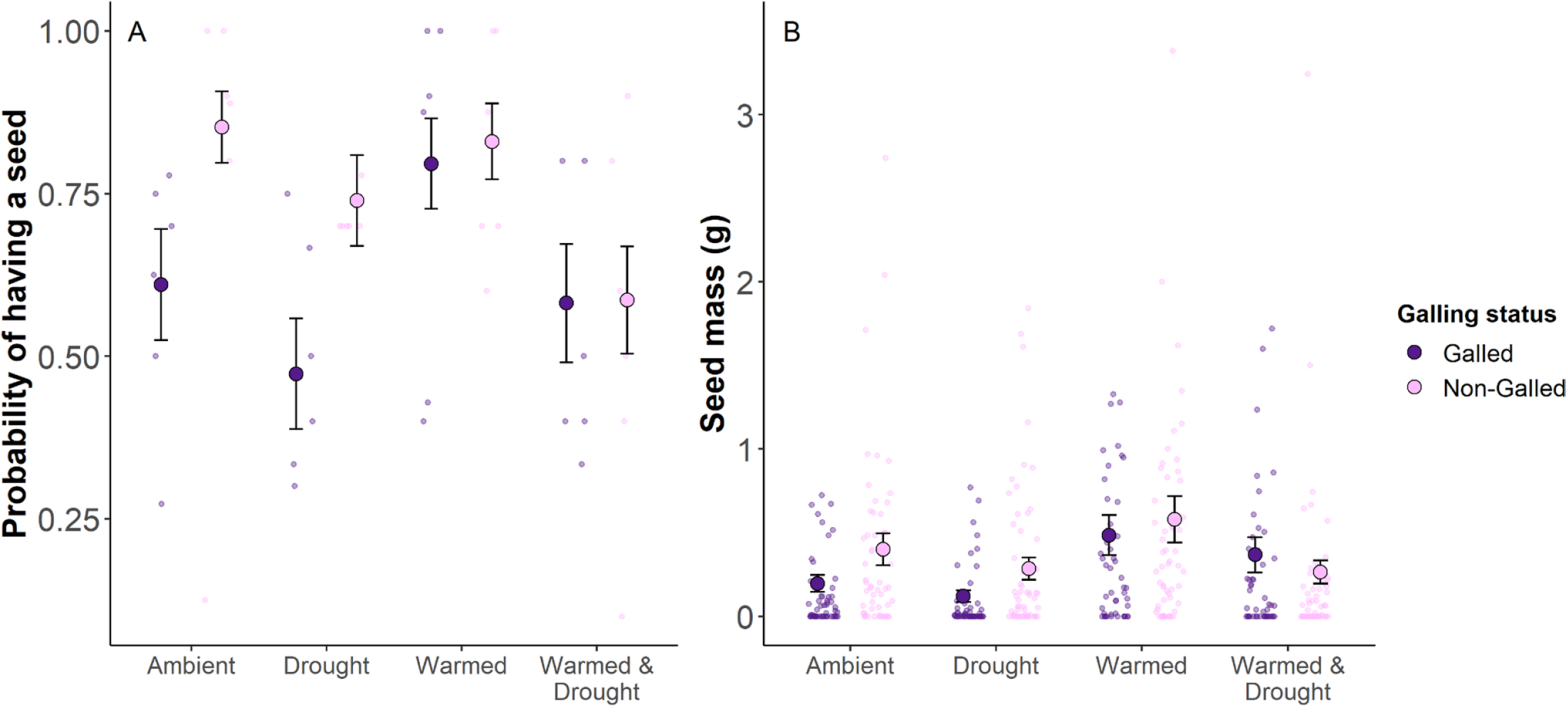
**A** The probability that a stem produced a seed, and **B** the mass of seeds produced (*g*). Data are presented for each climate treatment (ambient, drought, warmed, and warmed & drought). Data are split within each climate treatment for stems with galls (purple) and stems without galls (pink). Jittered points in the background represent **A** the plot-level probability of producing a seed, and **B** the seed mass for individual stems. Large points represent the model-estimated mean ± standard error. Sample sizes are in Table S1

Non-galled stems had a 0.24 higher probability of producing a seed compared to galled stems in the ambient treatment (*z*_inf_ = 2.67, *P* = 0.008), and a 0.27 higher probability in the drought treatment (*z*_inf_ = 2.79, *P* = 0.005; Fig. 4A). Galled stems in the drought treatment had a 0.32 lower probability of producing a seed compared to galled stems in the warmed treatment (*z*_inf_ = 2.69,* P* = 0.04). Non-galled stems in the ambient treatment had a 0.27 higher probability of producing seeds than non-galled stems in the warmed & drought treatment (*z*_inf_ = −2.54, *P* = 0.05). These results were consistent across both years of the study (Fig. S4).

#### Seed mass

When comparing only galled stems between climate treatments, we found that seed mass for galled stems in the ambient treatment was 0.29 *g* lower than galled stems in the warmed treatment (*z*_inf_ = −3.37, *P* = 0.004). Seed mass for galled stems in the drought treatment was 0.36 *g* lower than galled stems in the warmed treatment (*z*_inf_ = −3.72, *P* = 0.001), and 0.25 *g* lower than galled stems in the warmed and drought treatment (*z*_inf_ = −3.47, *P* = 0.003; Fig. 4B). When comparing seed mass between galled and non-galled stems, we found that galled-stem seed mass was 0.21 *g* lower in the ambient treatment (*z*_inf_ = −2.82, *P* = 0.005), and 0.16 *g* lower than non-galled stems in the drought treatment (*z*_inf_ = −3.10, *P* = 0.002). These results were consistent across both years of the study (Fig. S4).

### Gall biomass, chamber count, and chamber volume

**Fig. 5:**
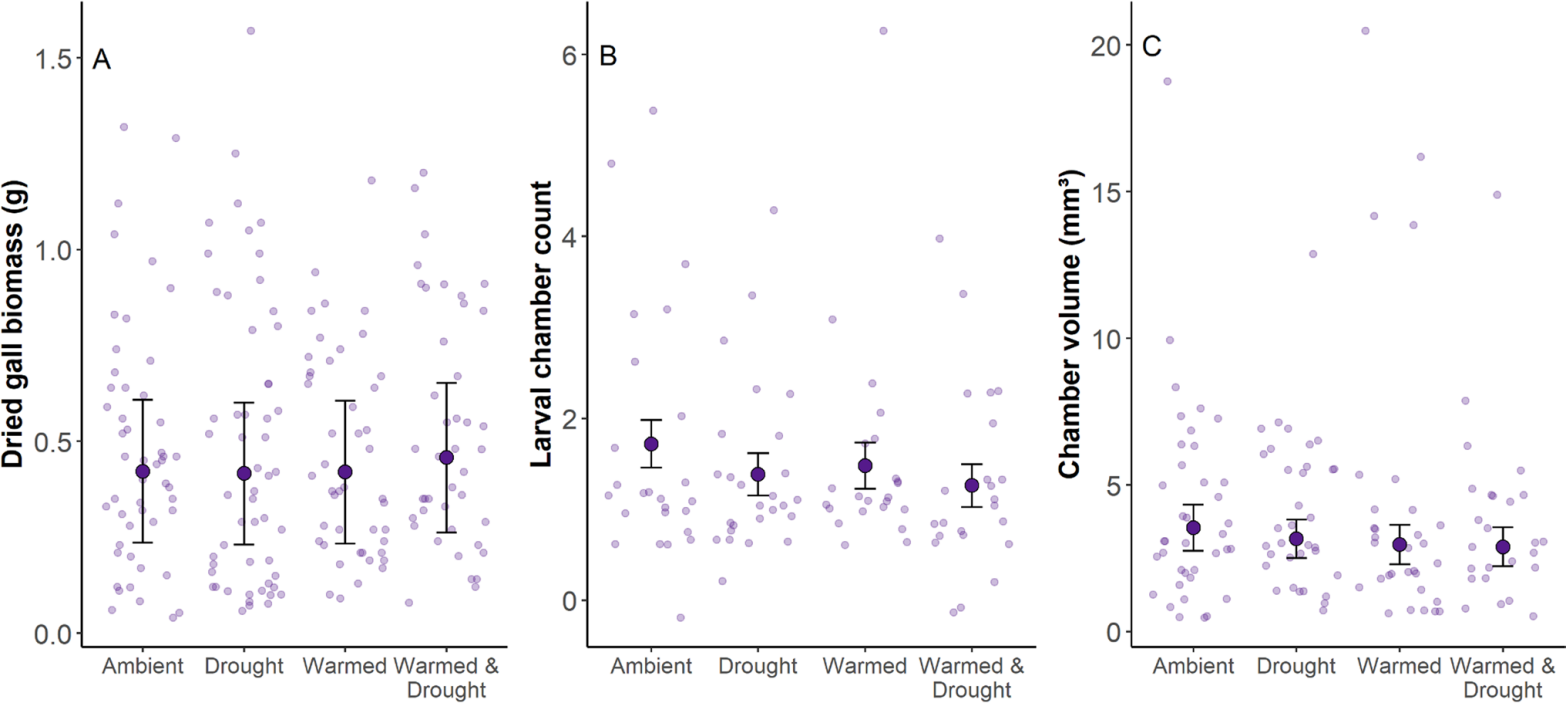
**A** Dried biomass of galls (*g*), **B** larval chamber count, and **C** larval chamber volume for stems from each climate treatment (ambient, drought, warmed, and warmed & drought). Jittered points in the background represent the individual, **A** dried gall biomass, **B** larval chamber count, and **C** larval chamber volume for individual stems in each treatment. Large points and error bars represent the model-estimated mean ± standard error. Sample sizes are in Table S1

There were no differences in gall biomass, chamber count, or chamber volume between climate treatments (Fig. 5, Table S5). The gall-biomass results were consistent between years, but gall biomass was overall lower in 2022 (Table S5, Fig. S5). We did not find a relationship between gall biomass and stem height or stem biomass (Fig. S6).

## Discussion

As climate change intensifies and creates more variable extremes, plants must cope with shifting abiotic conditions while maintaining defenses against negative biotic interactions. We investigated the effects of warming and drought on tall goldenrod stems with and without *R. solidaginis* galls. For all of our goldenrod measurements, galling modified the stem’s response to the climate treatment. Galling increased stem biomass in warmed conditions (Fig. 3A), and the effect of warming on stem height was stronger for galled stems (Fig. 3B). Furthermore, warming appears to offset the negative effects of galling for the probability of producing a seed and the seed mass, whereas drought alone appears to exacerbate negative galling effects on seed production probability (Fig. 4A). However, we did not find evidence for any climate effects on our three gall measurements (gall biomass, chamber count, or chamber volume; Fig. 5). These results demonstrate that climate warming reduces the negative effects of galling on plant fitness traits, but the galls’ traits are unaffected by the climate treatments in this system.

### Warming offsets the negative effect of galling for some plant traits

We found some support for our first hypothesis that warming will mitigate negative effects of galling on plant fitness traits. However, some plant traits showed a positive additive effect of warming and galling; for example, warming increased stem biomass for both galled and non-galled stems, but the effect was strongest for galled stems (Fig. 3A). Galling by *R. solidaginis* has been found to increase stem production (Hartnett and Abrahamson 1979), so warming and galling may be acting additively in this case. For other traits, we found evidence that warming reduces the negative effect of galling on plant fitness. This effect is especially evident in the seed mass and seed production probability results, wherein galling has a large negative effect on seed production probability in ambient and drought conditions, but this negative effect is mitigated under the warmed and warmed & drought treatments (Fig. 4A). Similarly, galled stems were shorter in ambient conditions, but under warming, this effect is reduced or reversed (Fig. 3B, S3). When *R. solidaginis* females oviposit eggs into the apical meristem of goldenrod, this halts stem growth and reallocates nutrients to the developing galls (Hartnett and Abrahamson 1979; Wise et al. 2006; Crutsinger et al. 2008). However, warming increases plant metabolism (Dusenge et al. 2019), which may offset the energy demand of galls and allow for further stem growth. There is also the possibility that the adult females may have been selecting for taller stems when ovipositing their eggs, which may lead to a bias towards galled stems being taller. These results show that under a warmer climate, the effects of galling on plant fitness may not be as severe if warming has the potential to mitigate galling’s negative effects. However, it is important to note that we did not comprehensively measure all facets of plant fitness and productivity (e.g., total number of seeds, leaf production, rhizome production, etc.) which *R. solidaginis* galling has been shown to affect (Hartnett and Abrahamson 1979; Wise et al. 2006; Crutsinger et al. 2008). Our results only demonstrate this mitigation effect for the traits shown, rather than for plant fitness as a whole.

### Galling and drought synergistically interact to decrease reproductive, but not vegetative traits

We found partial evidence for our second hypothesis that drought and galling will synergistically reduce plant fitness. This hypothesis was mainly supported in the seed analyses, where we found that the combination of drought and galling led to the lowest probability of producing a seed and the lowest seed mass (Fig. 4). We did not find evidence for this synergistic negative effect for stem height or biomass as the combination of galling and drought did not lead to significantly lower height or biomass compared to other treatment combinations (Fig. 3). Interestingly, although we see a strong synergistic negative effect of galling and drought on seed production probability, we again find evidence that warming mitigates this negative effect (Fig. 4A), which supports our third hypothesis. For example, galled and non-galled stems in the warmed-and-drought treatment have the same probability of producing a seed, whereas galling has a strong negative effect on seed production probability in the drought treatment, demonstrating that warming offsets the negative galling effects. The reallocation of resources to non-reproductive structures in response to galling and drought (Hartnett and Abrahamson 1979; Shibel and Heard 2016) may be offset by the increased metabolic activity of warming (Dusenge et al. 2019). However, we also found that non-galled stems in the ambient control plot had a higher probability of producing a seed than non-galled stems in the warmed-and-drought treatment, though the treatments did not differ in their average seed mass (Fig. 4). Warming and drought have been shown to reduce seed production (Rosenblatt 2018). Unlike this previous study, we did find a significant interactive effect of warming and drought, suggesting warming and drought may be acting synergistically to reduce reproduction in non-galled plants. The combination of warming and drought may reduce seed production because warming can exacerbate the negative impacts of drought by intensifying drought severity (Balting et al. 2021; Gebrechorkos et al. 2025), ultimately reducing the probability of producing a seed in non-galled stems.

### Galls are unaffected by climate treatments, but show high interannual variation

Climate treatments had no impact on *R. solidaginis* galls in our study (Fig. 5, S5), which goes against our fourth hypothesis. Warming and drought have been shown to reduce gallmaker fitness in other systems (Sumerford et al. 2000; Björkman 2000; Xi et al. 2016). One possible reason that we did not find a drought effect on galls in our study could be that the timing of our drought treatment was too late in the season or did not last long enough to impact gall development. Typically, *R. solidaginis* gall chambers begin to develop in mid-July, and galls reach their final size by July (Dorchin et al. 2009). Since galls had nearly reached full size by the time our drought started, they may have already stored the necessary water and nutrients to support larval development before drought was induced. Furthermore, adults of this species emerge from their galls in late September to early October (Dorchin et al. 2009). The timing of our gall dissections in late October 2021 meant that we were unable to monitor insect fitness or survival. It is possible that the measurement we chose to estimate larval fitness (larval chamber volume and count) is not a good proxy for this species. We did, however, find that the gall biomass was lower for all treatments in 2022 (Fig S5). Galls have been shown to be negatively affected by drought events (Sumerford et al. 2000), so the lower biomass may have been due to the experimental drought stress from 2021 or from the natural drought that occurred in spring 2021 (E. Parker and M. Hammond, pers. obs.). Sumerford et al. (2000) also found high interannual variability in gall size, so longer term studies would be necessary to determine whether the climate treatments had any effect on the galls of *R. solidaginis*.

### Why were vegetative traits of stems without galls not affected by our climate treatments?

Our climate treatments did not have strong effects on the height or biomass of stems without galls. Goldenrod has an extensive rhizomatous network belowground that can reach up to 2.5 m in diameter (Werner et al. 1980). Given the low levels of soil disturbance of the early-successional community within the KBS LTER early-successional fields (i.e., no tilling), it is possible that the individual stems (ramets) we measured are connected to stems outside of the treatments, which may be buffering the effects of our climate treatments. This may also mean that, due to our small plot size (1 m^2^), we may have stems that are clones of each other (e.g., multiple stems are a single-unique genet connected via rhizome) within the same plot. However, given the age (over 30 years) and presence of galling in our system, it is unlikely that these stems are connected, as goldenrods are more likely to “slough” rhizomal connections as the plants age (Maddox et al. 1989) and between ramets that are galled (McCrea and Abrahamson 1985; How et al. 1994). In fact, goldenrod stems in the KBS LTER early-successional communities show high genetic differentiation (M. Howard, pers. comm). Therefore, some genotypes may have been more resistant to the effects of warming (Springate and Kover 2014), which may be why we did not see strong effects of warming on individual stems. Additionally, within the KBS LTER, *S. altissima* has been the most dominant species by biomass in the early-successional treatments for over two decades, even in years with drought (Pérez et al. 2024). The stems in our system appear to be resilient to changes in precipitation, and therefore may not be strongly impacted by drought.

### Broader implications of goldenrod responses to climate changes and galling

With warmer temperatures predicted for both goldenrod’s native and invasive ranges (IPCC 2021), we may see an increase in goldenrod vegetative and reproductive fitness, leading to further declines in plant species diversity, which may have implications for broader ecosystem functioning. Without the mediation of insect herbivory, including specialized forms such as galling, goldenrod grows more densely in its introduced range (Jakobs et al. 2004). Insect populations worldwide are also facing rapid decline due to habitat loss, insecticides, and climate change (Sánchez-Bayo and Wyckhuys 2019; Rhodes 2019; Wagner et al. 2021; Outhwaite et al. 2022), which may further decrease insect herbivory pressure on goldenrod in its invasive range and lead to further expansion. We show here that under warming, many negative effects of galling are mitigated, which may allow goldenrod to further dominate ecosystems in its native range now and into the future.

## Conclusions

We found that galling modified goldenrod’s responses to climate treatments. Given goldenrod’s dominance and abundance in old-field ecosystems, understanding how this species will change with future climate regimes can provide key insight into future community dynamics. Here we show that negative effects of galling on plant fitness traits may be mitigated by climate warming, even under additional abiotic stressors such as drought, potentially allowing goldenrod to further dominate ecosystems. These results also broadly demonstrate the complexity of plant responses to climate change; because plants may be interacting with numerous biotic factors in the environment while simultaneously experiencing multiple abiotic stressors, it can be difficult to generalize plant trait responses to a given stressor. Moreover, multi-year studies that assess the simultaneous effects of varying biotic and abiotic treatments on plant traits can ultimately help inform the future of functional trait diversity, species interactions, and community composition.

## Supplementary Information

Below is the link to the electronic supplementary material.

Supplementary file1 (DOCX 2991 KB)

## Data Availability

Cleaned data (Parker et al. 2026a) is available in the Environmental Data Initiative’s EDI Data Portal at 10.6073/pasta/be138bef49e2040db4ce4e23b9cbced3. Raw data is available from the corresponding author upon reasonable request.

## Code availability

R code (Parker et al. 2026b) for cleaning, analyzing, and plotting our data is available on Zenodo at 10.5281/zenodo.18356227.
