# Supplementary material for: Climate warming and drought modify galling effects on tall goldenrod
Source: Oecologia. 2026 Apr 4;208(4):51. doi: 10.1007/s00442-026-05889-3 (PMC13048925; doi:10.1007/s00442-026-05889-3)
Supplement: Supplementary file 1 — Supplementary file1 (DOCX 2991 KB) [file 442_2026_5889_MOESM1_ESM.docx]

## Appendix S1: Plant taxonomy and community composition

### Plant identification and Reference Specimen

In the early years of the KBS-LTER, a conservative choice was made to identify the *Solidago* species within the LTER as Canada goldenrod (*Solidago canadensis*) because the flora references were not completely in consensus on nomenclature. In order to be consistent with nomenclature across years, *S. canadensis* was kept. More recently, a consensus within the floras (Reznicek et al. 2011) and additional expertise from plant biologists familiar with the early successional community have increased confidence in delineating the two species. The plants described in this study are identified as tall goldenrod (*Solidago altissima*). *S. altissima* was distinguished from *Solidago canadensis* objectively by measuring the length of multiple involucres from inflorescences on field plants and subjective observations on the location and degree of hairs on the underside of leaves. All specimens were determined to be *S. altissima*, with involucres averaging 3.1- 4.6mm in length, with hairs found in between veins on the underside of leaves (Reznicek et al. 2011).

We did not create an herbarium specimen to be used as a voucher for this study. However, Michigan State University (MSU) has an extensive herbarium collection including some digitized specimens. This collection was searched and a specimen that has been digitized was found that will serve as an excellent reference specimen (Catalog #: MSC0179791). The specimen was originally collected by W.T. Gillis on September 1st, 1976 in an “upland old-field” within approximately 1 mile of the future KBS LTER site. Originally it was identified as *Solidago canadensis*, but it was later re-examined by E.G.Voss in 1994 with the updated taxonomy of *Solidago altissima* L. The digitized specimen can be located using [Midwest Herbaria Portal](https://urldefense.com/v3/__https:/midwestherbaria.org/portal/collections/individual/index.php?occid=8058693&clid=0__;!!HXCxUKc!3_Uz7WHyOH7aqcwO2ACkAQDdnyidCnt6VwWelc9ZcIIFrEmEWPvH6CtLbRbVwfcstXqqoZ74P89oZysow0FNlC_qpQ$).

Secondarily, KBS has a limited herbarium collection onsite. The collection was searched for specimens to be used as a physical reference. Specimen #6451 is an excellent physical reference. It was collected by J. Fisher for K. Gross on September 16th, 1988 from an area adjacent, within a few hundred yards, of the eventual LTER site. Given the collection date and the start of the LTER in 1989, plus the proximity to the future LTER site, the collection was likely made originally to be used as a reference specimen. The species identification for this specimen is Solidago canadensis. However, the length of the involucres, the presence of hairs on the undersides of leaves and the lower stem all indicate an updated identification would be *Solidago altissima* (Reznicek et al. 2011)*.*

### KBS LTER T7 composition

This plant community is composed of non-agronomic forb, grass, and legume species, and is commonly referred to as an “old field.” The site contains 6 early successional community field replicates (1 ha, 87 x 105m). Each replicate has 5 stations utilized for monitoring and sampling of the plant community. Annual net primary productivity (ANPP) and species composition data have been collected annually since 1991 by harvesting aboveground biomass from 1 meter square areas (2m x 0.5m quad), sorting biomass to species, drying, and weighing the biomass. This sampling has resulted in a large, impressive data set covering 32 years (1991-2022; current year of data availability), 6 field replicates, and 5 stations per field replicate, equaling 960 potential unique sampling events. From 1991 to 2022, averaging within a year and within a field replicates’ 5-stations, descriptively the average ANPP for this community was 582.24 grams per meter square (min = 200.7, max = 762.84, standard deviation = 153.7, number of years = 30). ANPP was not harvested 1994-1996.

*Solidago altissima* is the dominant species in this community, both currently and since 1991. Of the 960 sampling events, 51 events were excluded due to the lack of biomass harvest and/or sorting to species. Of the remaining 849 events, 798 (94%) events had *S. altissima* present. Only 51 events had no *S. altissima* biomass harvested. Not only is *S. altissima* the most frequent species of this community, it is also the most dominant with the greatest productivity. Of the 849 events, *S. altissima* had the greatest biomass 472 times and the second greatest biomass 130 times. The next most dominant species is quackgrass (*Elymus repens*), with just 54 events as the most dominant and 43 events as the second most dominant species. On average per event, *S. altissima* had 220.9 grams of dried biomass per meter square (min = 0, max = 819.3, standard deviation = 177.1, number of events = 847), which corresponds to an average of 35.2% biomass for each event (min = 0, max = 97.2, standard deviation = 24.2, number of events = 847).

### References

Reznicek, A. A., E. G. Voss, and B. S. Walters. 2011, February. Michigan Flora. https://www.michiganflora.net/genus/Solidago.

Reference specimen from Michigan State University (MSC) Herbarium Collection, Catalog # MSC0179791. Digitized specimen available from [Midwest Herbaria Portal](https://midwestherbaria.org/portal/collections/individual/index.php?occid=8058693&clid=0)

###

## Supplementary tables

#### Table S1: Total count of plants in each climate treatment for the 2021 and 2022 seasons. Note that not all plants were used in every analysis due to individual variability, or the early senescence of some individuals.

|  | **Ambient** | | **Warming** | | **Drought** | | **Warming & Drought** | |  |
| --- | --- | --- | --- | --- | --- | --- | --- | --- | --- |
| **Galling Status** | Gall | No Gall | Gall | No Gall | Gall | No Gall | Gall | No Gall | **Total** |
| 2021 | 25 | 27 | 24 | 28 | 27 | 29 | 26 | 30 | 216 |
| 2022 | 31 | 27 | 23 | 29 | 30 | 29 | 20 | 27 | 216 |
| **Total** | 56 | 54 | 47 | 57 | 57 | 58 | 46 | 57 | **432** |

#### Table S2. A: Plant stem biomass linear mixed-model output testing for an effect of climate treatment, galling status, and the interaction between climate x galling on stem biomass. B: Pairwise contrasts of differences between climate treatments based on galling status. C: Pairwise contrasts of differences between galling status based on climate treatment.

| **(A) Treatment** | **Sum Sq** | **Mean Sq** | **NumDF** | **DenDF** | **F-Value** | ***P*-Value** |
| --- | --- | --- | --- | --- | --- | --- |
| Climate | 5.04 | 1.68 | 3 | 14.0 | 8.86 | **0.002** |
| Galling | 2.90 | 2.90 | 1 | 411 | 15.3 | **0.0001** |
| Climate:Galling | 1.95 | 0.65 | 3 | 411 | 3.43 | **0.017** |
| **(B) Contrast** | **Galling Status** | **Estimate** | **SE** | **DF** | **T-Ratio** | **P-Value** |
| Ambient - Drought | Galled | -0.09 | 0.13 | 22.6 | -0.64 | 0.92 |
| Ambient - Warmed | Galled | -0.43 | 0.12 | 31.7 | -3.72 | **0.004** |
| Ambient - Warmed Drought | Galled | -0.53 | 0.14 | 25.6 | -3.87 | **0.003** |
| Drought - Warmed | Galled | -0.34 | 0.14 | 24.6 | -2.54 | 0.08 |
| Drought - Warmed Drought | Galled | -0.45 | 0.12 | 32.9 | -3.82 | **0.003** |
| Warmed - Warmed Drought | Galled | -0.10 | 0.14 | 27.6 | -0.73 | 0.88 |
| Ambient - Drought | Non-galled | -0.03 | 0.13 | 22.8 | -0.26 | 0.99 |
| Ambient - Warmed | Non-galled | -0.27 | 0.11 | 28.5 | -2.43 | 0.09 |
| Ambient - Warmed Drought | Non-galled | -0.17 | 0.13 | 22.8 | -1.27 | 0.59 |
| Drought - Warmed | Non-galled | -0.24 | 0.13 | 22.2 | -1.80 | 0.30 |
| Drought - Warmed Drought | Non-galled | -0.14 | 0.11 | 27.4 | -1.2 | 0.62 |
| Warmed - Warmed Drought | Non-galled | 0.10 | 0.13 | 22.1 | 0.78 | 0.86 |
|  |  |  |  |  |  |  |
|  |  |  |  |  |  |  |
| **(C) Contrast** | **Climate** | **Estimate** | **SE** | **DF** | **T-Ratio** | **P-Value** |
| Galled - Non-galled | Ambient | 0.02 | 0.08 | 412 | 0.29 | 0.77 |
| Galled - Non-galled | Drought | 0.08 | 0.08 | 410 | 0.92 | 0.36 |
| Galled - Non-galled | Warmed | 0.18 | 0.09 | 414 | 2.07 | **0.04** |
| Galled - Non-galled | Warmed Drought | 0.39 | 0.09 | 424 | 4.31 | **<0.0001** |

#### Table S3. A: Plant height linear mixed-model output testing for an effect of climate treatment, galling status, and the interaction between climate x galling on height. B: Pairwise contrasts of differences between climate treatments based on galling status. C: Pairwise contrasts of differences between galling status based on climate treatment.

| **(A) Treatment** | **Sum Sq** | **Mean Sq** | **NumDF** | **DenDF** | **F-Value** | ***P*-Value** |
| --- | --- | --- | --- | --- | --- | --- |
| Climate | 1.05 | 0.35 | 3 | 14.2 | 8.03 | **0.002** |
| Galling | 0.06 | 0.06 | 1 | 409 | 1.44 | 0.23 |
| Climate:Galling | 0.45 | 0.15 | 3 | 410 | 3.42 | **0.02** |
| **(B) Contrast** | **Galling Status** | **Estimate** | **SE** | **DF** | **T-Ratio** | **P-Value** |
| Ambient - Drought | Galled | -0.03 | 0.08 | 20.0 | -0.31 | 0.99 |
| Ambient - Warmed | Galled | -0.24 | 0.07 | 22.6 | -3.39 | **0.01** |
| Ambient - Warmed Drought | Galled | -0.30 | 0.08 | 21.6 | -3.54 | **0.009** |
| Drought - Warmed | Galled | -0.22 | 0.08 | 21.1 | -2.63 | 0.07 |
| Drought - Warmed Drought | Galled | -0.27 | 0.07 | 22.9 | -3.74 | **0.005** |
| Warmed - Warmed Drought | Galled | -0.05 | 0.08 | 22.6 | -0.61 | 0.93 |
| Ambient - Drought | Non-galled | 0.03 | 0.08 | 20.1 | 0.36 | 0.98 |
| Ambient - Warmed | Non-galled | -0.17 | 0.07 | 21.1 | -2.41 | 0.10 |
| Ambient - Warmed Drought | Non-galled | -0.11 | 0.08 | 20.1 | -1.40 | 0.52 |
| Drought - Warmed | Non-galled | -0.20 | 0.08 | 19.8 | -2.45 | 0.10 |
| Drought - Warmed Drought | Non-galled | -0.14 | 0.07 | 20.6 | -2.02 | 0.21 |
| Warmed - Warmed Drought | Non-galled | 0.06 | 0.08 | 19.8 | 0.71 | 0.89 |
|  |  |  |  |  |  |  |
|  |  |  |  |  |  |  |
| **(C) Contrast** | **Climate** | **Estimate** | **SE** | **DF** | **T-Ratio** | **P-Value** |
| Galled - Non-galled | Ambient | -0.10 | 0.04 | 412 | -2.54 | **0.01** |
| Galled - Non-galled | Drought | -0.05 | 0.04 | 411 | -1.20 | 0.23 |
| Galled - Non-galled | Warmed | -0.03 | 0.04 | 413 | -0.70 | 0.49 |
| Galled - Non-galled | Warmed Drought | 0.08 | 0.04 | 418 | 1.89 | **0.06** |

####

#### Table S4. A: Overall zero-inflated gamma model output testing for an effect of climate treatment, galling status, and the interaction between climate x galling on seed mass/probability. B: Pairwise contrasts of differences in the probability of producing a seed between climate treatments based on galling status. C: Pairwise contrasts of differences in the probability of producing a seed between galling status based on climate treatment. D: Pairwise contrasts of differences in seed mass between climate treatments based on galling status. E: Pairwise contrasts of differences in seed mass between galling status based on climate treatment.

| **(A) Treatment** | **ChiSq** | **DF** | **P-Value** |  |  |  |
| --- | --- | --- | --- | --- | --- | --- |
| Climate | 18.1 | 3 | **0.003** |  |  |  |
| Galling | 7.71 | 1 | **0.005** |  |  |  |
| Climate:Galling | 11.0 | 3 | **0.012** |  |  |  |
| **(B) Contrast** | **Galling Status** | **Estimate** | **SE** | **DF** | **Z-Ratio** | ***P*-Value** |
| Ambient - Drought | Galled | -0.56 | 0.50 | Inf | -1.12 | 0.67 |
| Ambient - Warmed | Galled | 0.91 | 0.48 | Inf | 1.90 | 0.22 |
| Ambient - Warmed Drought | Galled | -0.12 | 0.52 | Inf | -0.23 | 1.00 |
| Drought - Warmed | Galled | 1.47 | 0.55 | Inf | 2.69 | **0.04** |
| Drought - Warmed Drought | Galled | 0.44 | 0.42 | Inf | 1.03 | 0.73 |
| Warmed - Warmed Drought | Galled | -1.03 | 0.57 | Inf | -1.81 | 0.26 |
| Ambient - Drought | Non-galled | -0.71 | 0.57 | Inf | -1.26 | 0.59 |
| Ambient - Warmed | Non-galled | -0.17 | 0.52 | Inf | -0.32 | 0.99 |
| Ambient - Warmed Drought | Non-galled | -1.40 | 0.55 | Inf | -2.54 | **0.05** |
| Drought - Warmed | Non-galled | 0.54 | 0.55 | Inf | 1.00 | 0.75 |
| Drought - Warmed Drought | Non-galled | -0.69 | 0.41 | Inf | -1.69 | 0.32 |
| Warmed - Warmed Drought | Non-galled | -1.24 | 0.53 | Inf | -2.32 | 0.09 |
|  |  |  |  |  |  |  |
| **(C) Contrast** | **Climate** | **Estimate** | **SE** | **DF** | **Z-Ratio** | ***P*-Value** |
| Galled - Non-galled | Ambient | 1.30 | 0.49 | Inf | 2.67 | **0.008** |
| Galled - Non-galled | Drought | 1.15 | 0.41 | Inf | 2.79 | **0.005** |
| Galled - Non-galled | Warmed | 0.22 | 0.51 | Inf | 0.44 | 0.66 |
| Galled - Non-galled | Warmed Drought | 0.02 | 0.42 | Inf | 0.05 | 0.96 |
| **(D) Contrast** | **Galling Status** | **Estimate** | **SE** | **DF** | **Z-Ratio** | ***P*-Value** |
| Ambient - Drought | Galled | 0.48 | 0.37 | Inf | 1.29 | 0.56 |
| Ambient - Warmed | Galled | -0.91 | 0.27 | Inf | -3.37 | **0.004** |
| Ambient - Warmed Drought | Galled | -0.63 | 0.38 | Inf | -1.67 | 0.33 |
| Drought - Warmed | Galled | -1.39 | 0.37 | Inf | -3.72 | **0.001** |
| Drought - Warmed Drought | Galled | -1.11 | 0.32 | Inf | -3.47 | **0.003** |
| Warmed - Warmed Drought | Galled | 0.28 | 0.37 | Inf | 0.74 | 0.88 |
| Ambient - Drought | Non-galled | 0.34 | 0.33 | Inf | 1.03 | 0.72 |
| Ambient - Warmed | Non-galled | -0.37 | 0.24 | Inf | -1.55 | 0.39 |
| Ambient - Warmed Drought | Non-galled | 0.41 | 0.35 | Inf | 1.18 | 0.69 |
| Drought - Warmed | Non-galled | -0.71 | 0.33 | Inf | -2.14 | 0.13 |
| Drought - Warmed Drought | Non-galled | 0.07 | 0.26 | Inf | 0.27 | 0.99 |
| Warmed - Warmed Drought | Non-galled | 0.78 | 0.34 | Inf | 2.29 | 0.10 |
| **(E) Contrast** | **Climate** | **Estimate** | **SE** | **DF** | **Z-Ratio** | ***P*-Value** |
| Galled - Non-galled | Ambient | -0.72 | 0.25 | Inf | -2.82 | **0.005** |
| Galled - Non-galled | Drought | -0.86 | 0.28 | Inf | -3.10 | **0.002** |
| Galled - Non-galled | Warmed | -0.18 | 0.25 | Inf | -0.72 | 0.47 |
| Galled - Non-galled | Warmed Drought | 0.33 | 0.28 | Inf | 1.15 | 0.25 |

#### Table S5. A: Gall biomass linear mixed-model output testing for an effect of climate treatment and year on biomass. B: Gall chamber count linear mixed-model output testing for an effect of climate treatment on the number of chambers. C: Gall volume linear mixed-model output testing for an effect of climate treatment on gall volume.

| **(A) Treatment** | **Sum Sq** | **Mean Sq** | **DF** | **F-Value** | ***P*-Value** |
| --- | --- | --- | --- | --- | --- |
| Climate | 0.020 | 0.007 | 3 | 0.21 | 0.86 |
| **(B) Treatment** | **Sum Sq** | **Mean Sq** | **DF** | **F-Value** | ***P*-Value** |
| Climate | 1.87 | 0.63 | 3 | 0.62 | 0.60 |
| **(C) Treatment** | **Sum Sq** | **Mean Sq** | **DF** | **F-Value** | ***P*-Value** |
| Climate | 0.27 | 0.09 | 3 | 0.32 | 0.81 |

## Supplementary Figures

**
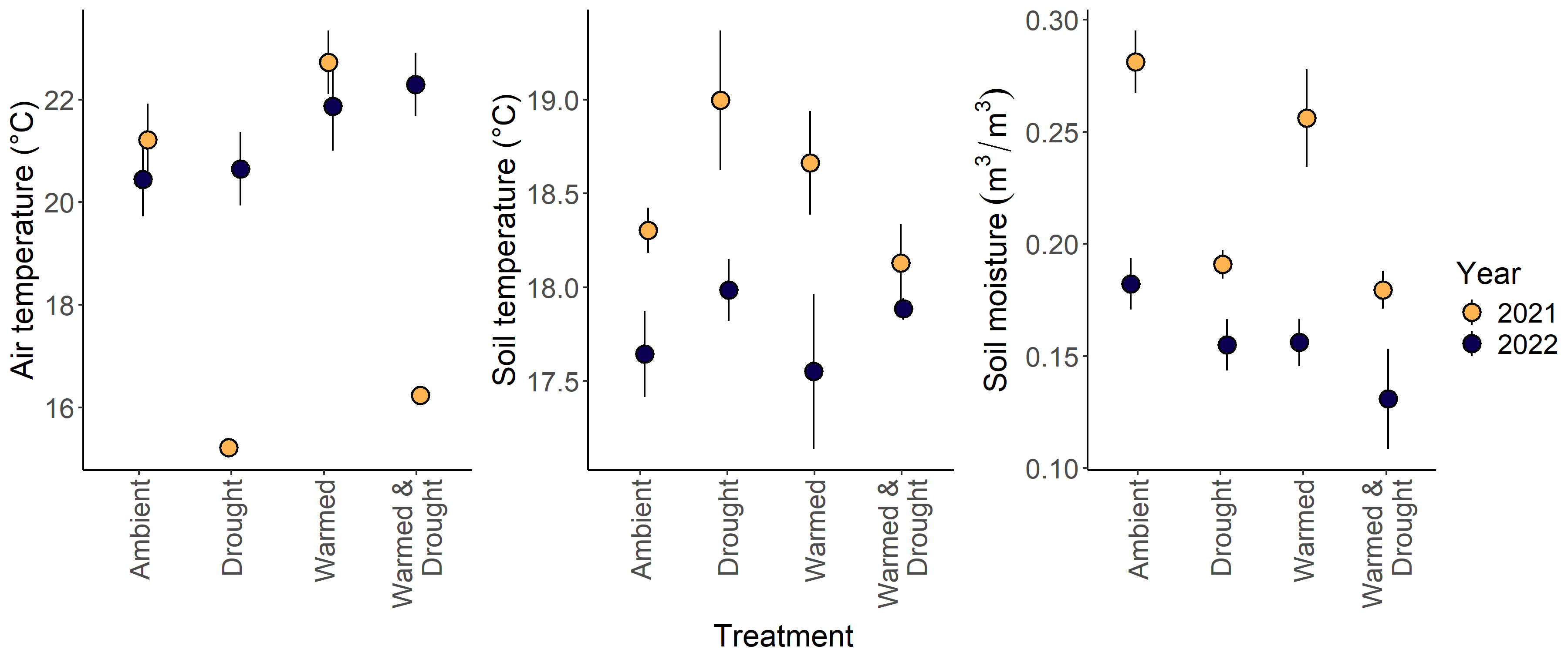
**

#### Figure S1. Air temperature (°C) at 1m aboveground, soil temperature (°C) at soil surface, and soil moisture (m^3^/m^3^) at an integrated depth of 25cm belowground across all climate treatments (irrigated, ambient, warmed, drought, and warmed & drought) in both years of the experiment (2021 and 2022). 2021 air temperature sensors for drought and warmed & drought were deployed in September, rather than July, leading to reduced air temperatures for those treatments (the 2021 drought and warmed & drought air temperature data were removed for analyses in the main paper).

###
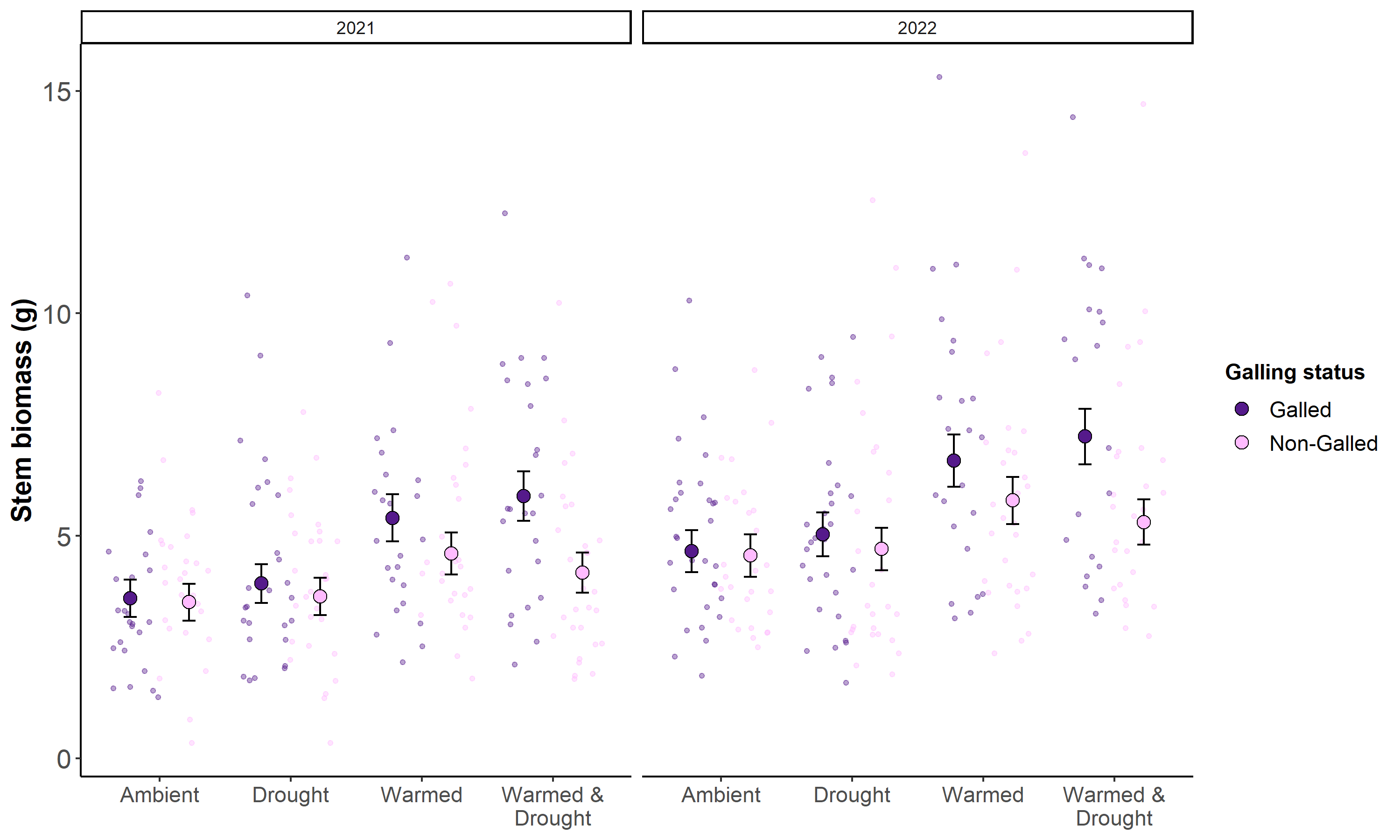


#### Figure S2. Stem biomass (g) across all climate treatments, split between plant galling status and year. Jittered points in the background represent plant stem biomass for individual plants from each treatment. Large points and error bars represent the model estimated mean ± standard error.

####
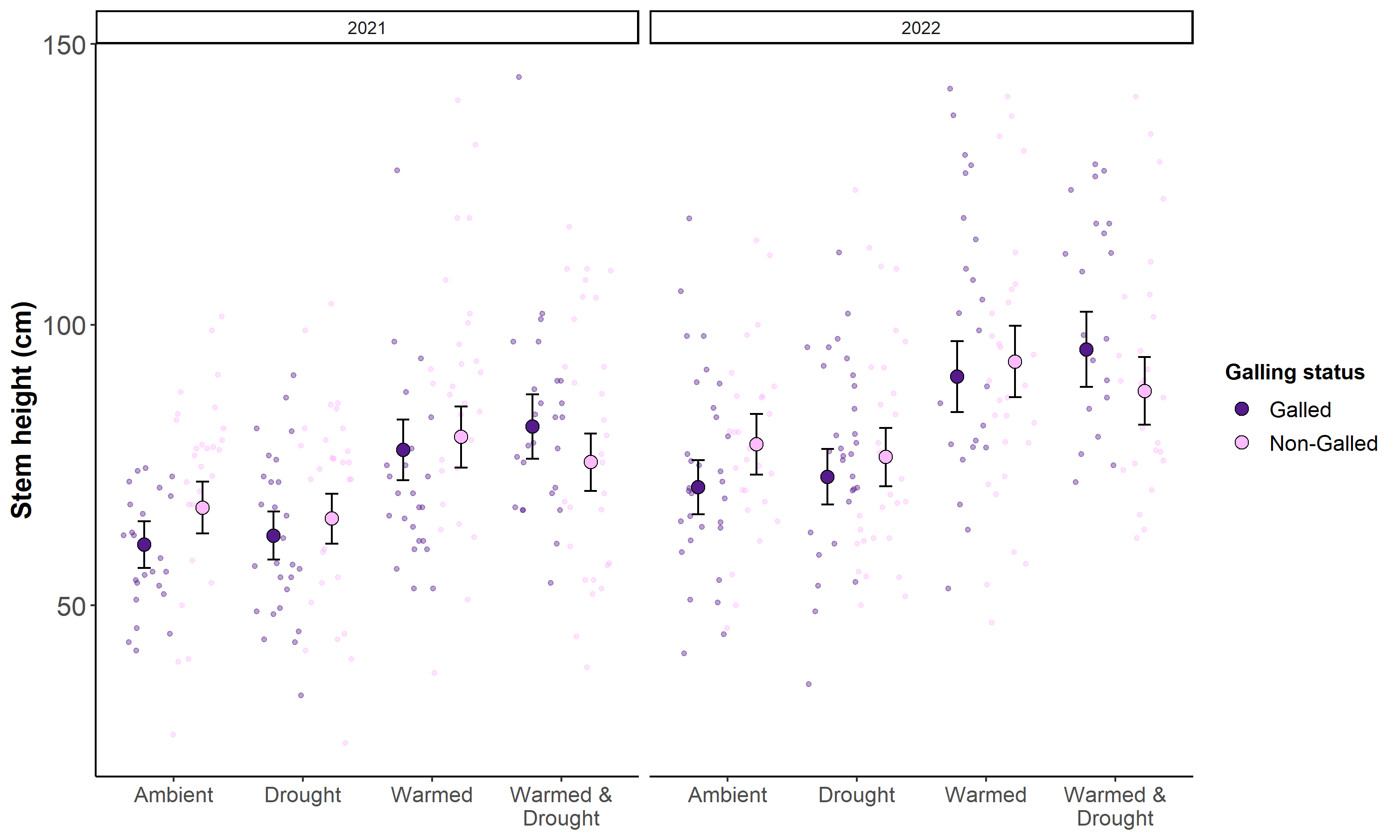
Figure S3. Stem height (cm) across all climate treatments, split between plant galling status and year. Jittered points in the background represent plant height for individual plants from each treatment. Large points and error bars represent the model estimated mean ± standard error.


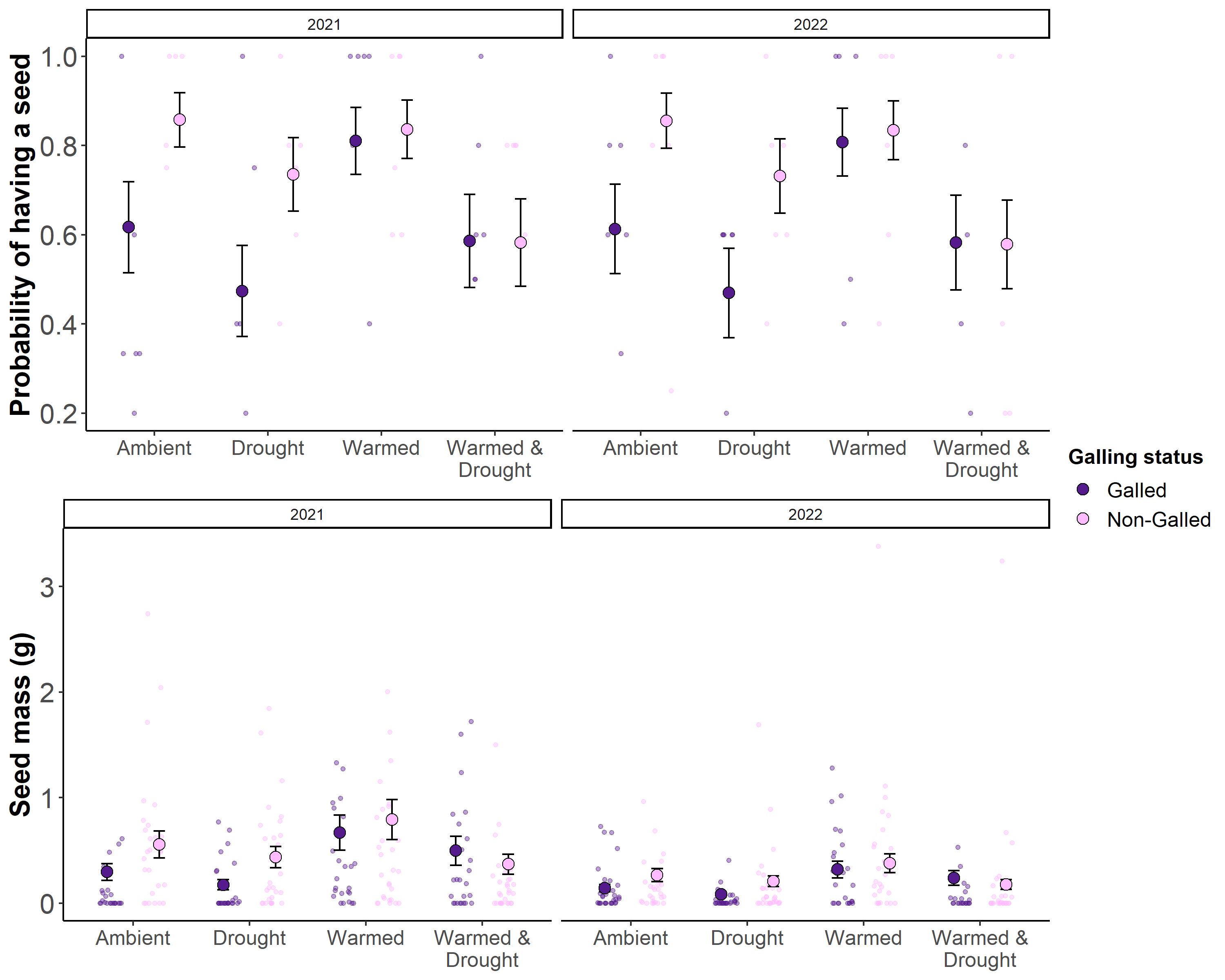


#### Figure S4. The probability that a stem produced a seed and average seed mass (g) across all climate treatments, split between plant galling status and year. Jittered points in the background represent the subplot-level probability of producing a seed, and the seed mass for individual stems. Large points and error bars represent the model estimated mean ± standard error.


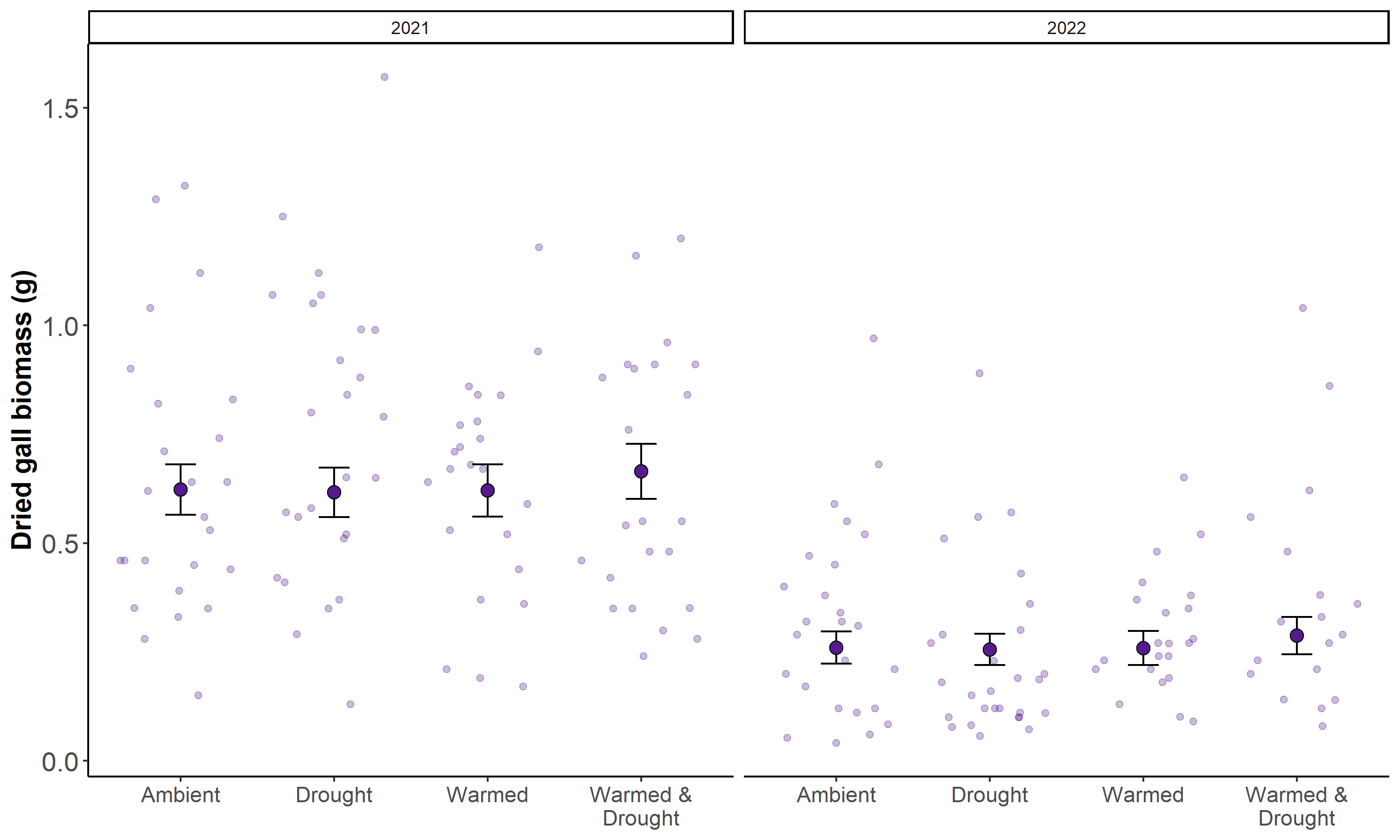


#### Figure S5. Dried gall biomass (g) across all climate treatments, split between year. Jittered points in the background represent dried gall biomass for individual stems from each treatment. Large points and error bars represent the model estimated mean ± standard error.


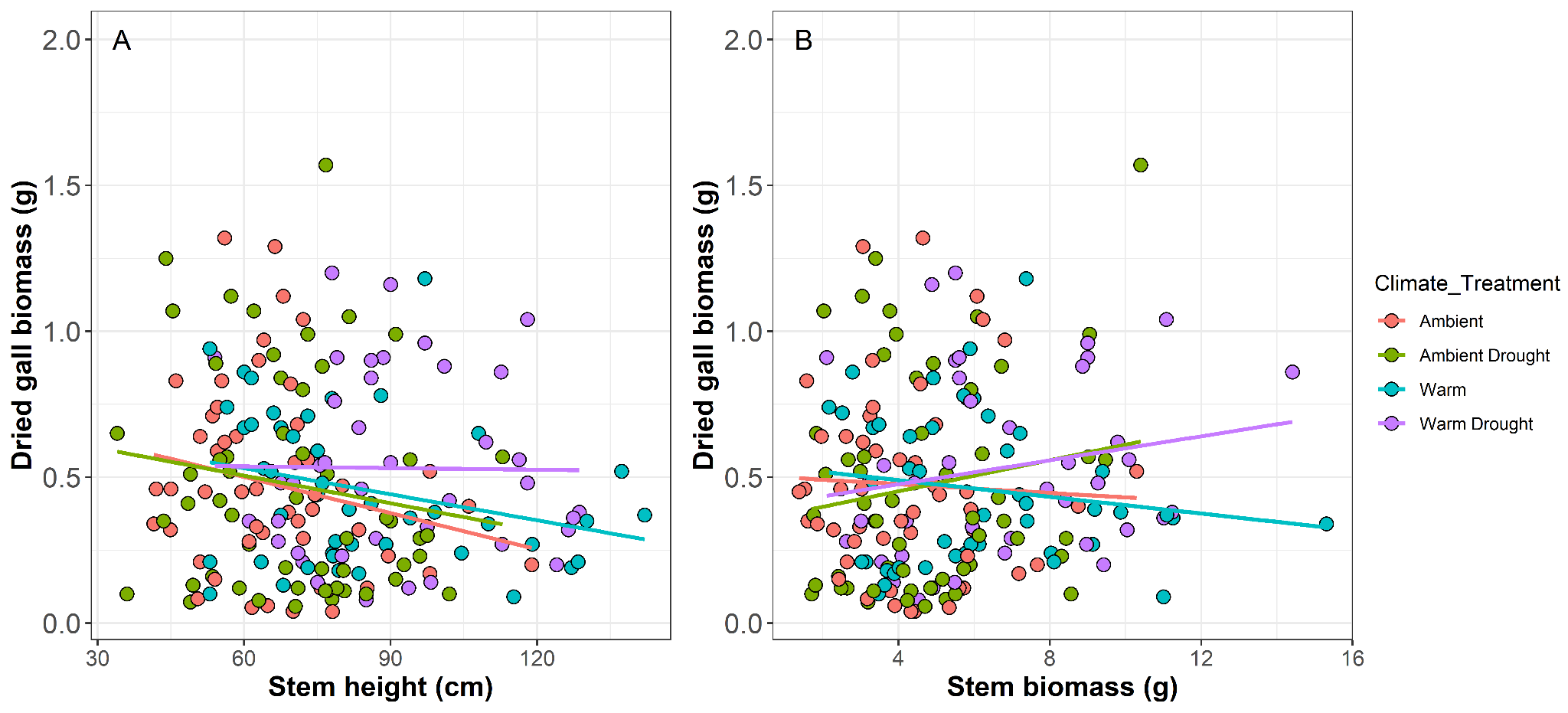


#### Figure S6. Dried gall biomass (g) compared to (A) stem height (cm) and (B) stem biomass (g). Data are split between different climate treatments (orange = ambient, green = drought, blue = warmed, purple = warmed & drought). Dried gall biomass was not predicted by stem height (F_1,190_ = 2.892, p = 0.09) or stem biomass (F_1,190_ = 0.761, p = 0.3841)
